# Trends and patterns of antimicrobial resistance among *Staphylococcus* aureus isolated from various clinical specimens: a national laboratory-based AMR surveillance, 2020–2024

**DOI:** 10.1038/s41598-026-51962-5

**Published:** 2026-05-09

**Authors:** Zeleke Ayenew, Meseret Aseffa Oda, Semira Ebrahim Geleto, Tewodros Minwuyelet Anteneh, Etsehiwot Adamu Tsegaye, Yonas Mekonnen Gebeyehu, Dejenie Shiferaw Teklu, Degefu Beyene Gobena, Amete Mihret Teshale, Gebrie Alebachew Belete, Surafel Fentaw Dinku, Tesfa Addis Kefale, Elias Seyoum Derebe, Abera Abdeta Kitaba, Dawit Assefa Wendifraw, Ebisa Fekede, Bruke Gezhagene Birhanu, Mheret Tesfaye Gobena, Addis Abebe, Degefe Habte, Senahara Korsa Wake, Dawit Sekata Duressa, Jemal Mohammed Hussien, Gemechis Bulti Tura, Eshetu Gadisa, Dereje Mamuye Gebretsadik, Daniel Dejene Wondimagegnehu, Daniel Demissie Gebremariam, Birhan Moges Ejigu, Bethelhem Getu Bekele, Estifanos Tsigie, Degu Ashagrie Alemu, Mezgebu Nega Bizuneh, Yesewbelay Minale Negesse, Rajiha Abubeker Ibrahim, Abebe Aseffa Negeri, Getachew Tollera Eticha, Gemechu Tadese Leta, Negga Asamene Abera, Eyasu Tigabu Seyoum

**Affiliations:** 1 Ethiopian Public Health Institute, PO Box 1242, Addis Ababa, Ethiopia; 2 Felege Hiwot Comprehensive Specialized Hospital, Bahir Dar, Ethiopia

**Keywords:** Trend, *S. aureus*, MRSA, Sentinel site, AMR surveillance, Ethiopia

## Abstract

Methicillin-resistant *Staphylococcus aureus* (MRSA) is a global public health concern responsible for a wide range of infections. In Ethiopia, patterns and trends of MRSA in various clinical specimens are limited. Therefore, this study aimed to determine the trend and pattern of *S. aureus* isolated from 16 AMR surveillance sentinel sites. Laboratory-based AMR surveillance data from 2020 to 2024 was collected from various clinical specimens. Culture and antimicrobial susceptibility testing were performed using standard microbiology techniques. The data were analyzed using WHONET 2025 software version 25.15.5 and SPSS version 22. A total of 4,243 *S. aureus* isolates were analyzed in 16 AMR sentinel sites; most of them were from health facilities in Addis Ababa (46.6%). The trend of penicillin resistance among tested isolates showed persistently high (~ 90%) between 2020 and 2024. The trend of MRSA showed a noticeable slight decline from 37% in 2021 and 2022 to 30% in the 2024 period, and its overall prevalence was 32.2% (95% CI 30.3, 34.2). There has been a slight declining trend of MRSA since 2022 and fluctuating resistance rates in routinely tested antimicrobials. The susceptibility of isolates to last-resort antibiotics remains promising for treatment. The significant presence of MRSA in participating hospitals warrants continuous surveillance and strengthens the implementation of infection prevention and control measures.

## Introduction

*Staphylococcus aureus* is a common bacterium frequently isolated from diverse clinical samples and is responsible for a broad range of illnesses, including furunculosis, cellulitis, abscesses, pyoderma, toxic shock syndrome, staphylococcal scalded skin syndrome, endocarditis, septicemia, and pneumonia^1^. The organism has developed resistance following the first introduction of penicillin in the early 1940s due to the selective pressure of resistant strains. For example, *Streptococcus pyogenes* strains are still susceptible to penicillin, while *S. aureus* strains are highly resistant to it^2,3^.

Since 2017, MRSA has been classified by the World Health Organization (WHO) as a high-priority pathogen on the global list of antibiotic-resistant bacteria^4^. Broadly, MRSA infections are categorized into two main types: hospital-associated MRSA (HA-MRSA) infections and community-associated MRSA (CA-MRSA) infections^5^.

MRSA strains associated with infections in hospitals, communities, and other settings exhibit distinct variations in their phenotypic and genetic backgrounds. Hospital-associated MRSA (HA-MRSA) strains are typically multidrug-resistant, with resistance extending beyond β-lactams, mainly responsible for invasive healthcare-associated infections such as bloodstream infections, surgical-site infections, device-related infections, and nosocomial pneumonia; genetically, they commonly harbor large *SCCmec* types I–III and are linked to international hospital clones, including CC5, CC8, CC22, CC30, and CC45, with lower prevalence of Panton-Valentine leukocidin (PVL) genes. In contrast, community-associated MRSA (CA-MRSA) strains tend to display less extensive antimicrobial resistance, often remaining susceptible to many non-β-lactam agents, and are frequently associated with skin and soft tissue infections (SSTIs) as well as necrotizing pneumonia in apparently healthy individuals. These strains usually carry smaller *SCCmec* types IV or V and are often PVL-positive**,** a feature contributing to tissue necrosis and enhanced virulence. This group includes notable epidemic clones such as USA300 (CC8) and, historically, USA400^6,7^.

Resistance can either develop by horizontal transfer of resistance determinants encoded by mobile genetic elements through plasmids, transposons, and the staphylococcal cassette chromosome or by mutations in chromosomal genes. Horizontally acquired resistance can occur by one of the following mechanisms: (i) enzymatic drug modification and inactivation, (ii) enzymatic modification of the drug binding site, (iii) drug efflux, (iv) bypass mechanisms involving acquisition of a novel drug-resistant target, and (v) displacement of the drug to protect the target^8^.

MRSA is generally resistant to cephalosporins and β-lactam antibiotics, which include penicillin, methicillin, and oxacillin. β-lactam inhibits the development of bacteria by preventing the production of the cell wall. MRSA usually gets around the effects of β-lactams by producing β-lactamase and altering the binding site for cell wall formation. The widely recognized therapeutic method for treating MRSA infection involves the use of several antibiotics, such as vancomycin and teicoplanin^9^.

The most prescribed antibiotic for MRSA infections is vancomycin. However, clinical MRSA isolates with decreased sensitivity to vancomycin have lately surfaced because of the growing usage of the antibiotic. Furthermore, vancomycin-intermediate *S. aureus* (VISA) infections and heterogeneous vancomycin-intermediate *S. aureus* (h-VISA) infections have poor clinical outcomes^10^. Other alternative treatment options include highly active monotherapies such as linezolid, daptomycin, and ceftaroline, as well as combination therapies of vancomycin with β-lactams, quinolones, or lipopeptides to exploit potential synergistic effects^11^.

In different studies of Ethiopia, vancomycin-resistant *S. aureus* is overestimated because disk diffusion AST methods are used incorrectly^12^. However, the real picture of VRSA is when it is tested in the lab using MIC or E-test-based VRSA testing, as CLSI^13^ suggests. To improve compliance and standardize testing methods at all AMR surveillance sites and beyond, ongoing mentoring and supervision have been implemented.

There is a lack of national evidence regarding the prevalence of MRSA and its trend of susceptibility to antimicrobial agents over time, which is a significant concern in Ethiopia. This study aimed to evaluate the trend and antimicrobial susceptibility pattern of *S. aureus* isolates in clinical specimens over a five-year period from AMR surveillance sentinel sites.

## Methods and materials

### Study design, study population, and study setting

This study employed a retrospective study design to analyze collected data on *Staphylococcus aureus* isolates retrieved from WHONET and archived isolates in the national biobank repository system. The isolates were obtained from clinical specimens in patients of all age groups across multiple healthcare facilities designated as AMR sentinel sites over a period from 2020 to 2024 through a large Ethiopian AMR surveillance system.

**Fig. 1:**
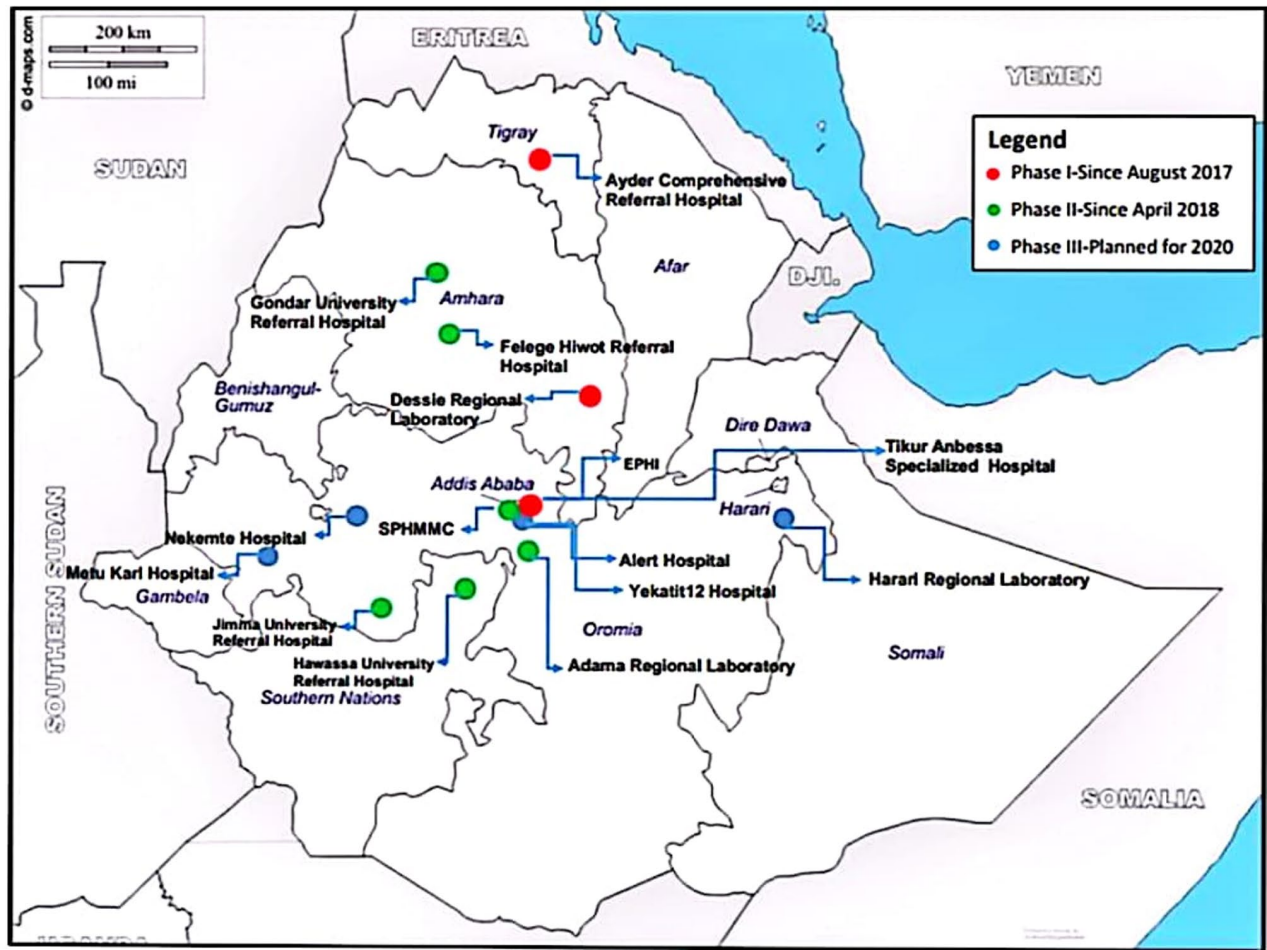
Spatiotemporal distribution of the first three phases of AMR sentinel sites in Ethiopia. Phase I: Tikur Anbessa Specialized Hospital, Addis Ababa; Ayder Referral and University Hospital, Tigray; Dessie Regional Laboratory, Amhara; National Clinical Bacteriology and Mycology Reference Laboratory, Addis Ababa. Phase II- St. Paul Millennium Medical College’s Hospital, Addis Ababa; Jimma University Specialized Hospital (later changed to Jimma Medical Center), Oromia; Felegehiwot Comprehensive Specialized Hospital, Amhara; Gondar University Specialized Hospital, Amhara; Hawassa Referral Hospital, SNNP. Phase III- Yekatit 12 Hospital, Addis Ababa; Zewuditu Hospital, Addis Ababa; Metu Karl Hospital, Oromia; Nekemt Regional, Oromia; Hiwot Fana Hospital, Harar; ALERT Hospital, Addis Ababa.

According to the national AMR surveillance system, selection of sites was a phased approach that recruits sentinel sites based on their availability, functional microbiology, committed laboratory staff and management, infrastructure, high sample volume, and the presence of antimicrobial stewardship (AMS) and infection prevention and control (IPC) committees after being assessed with a standard checklist^14^. Until the beginning of 2020, there were a total of 16 AMR sites that had undergone three phases: phase I (4 hospitals), phase II (5 hospitals), and phase III (7 hospitals), in which the majority of them (6 hospitals) had a large sample flow and a major referral site with better capacity in the capital, Addis Ababa. Most of the chosen facilities were for referrals and tertiary care hospitals that serve the general population of the country, where patients from rural areas and other regions go for better care (Fig. 1).

The National Clinical Bacteriology and Mycology Reference Laboratory under the Ethiopian Public Health Institute (EPHI) serves as a reference laboratory equipped with BD Phoenix and Vitek® 2 (bioMérieux) automated systems and real-time PCR in addition to standard microbiological culture methods to identify and confirm isolates received from sentinel sites regularly. The laboratory has maintained its full accreditation since 2017.

### Data extraction

**Fig. 2:**
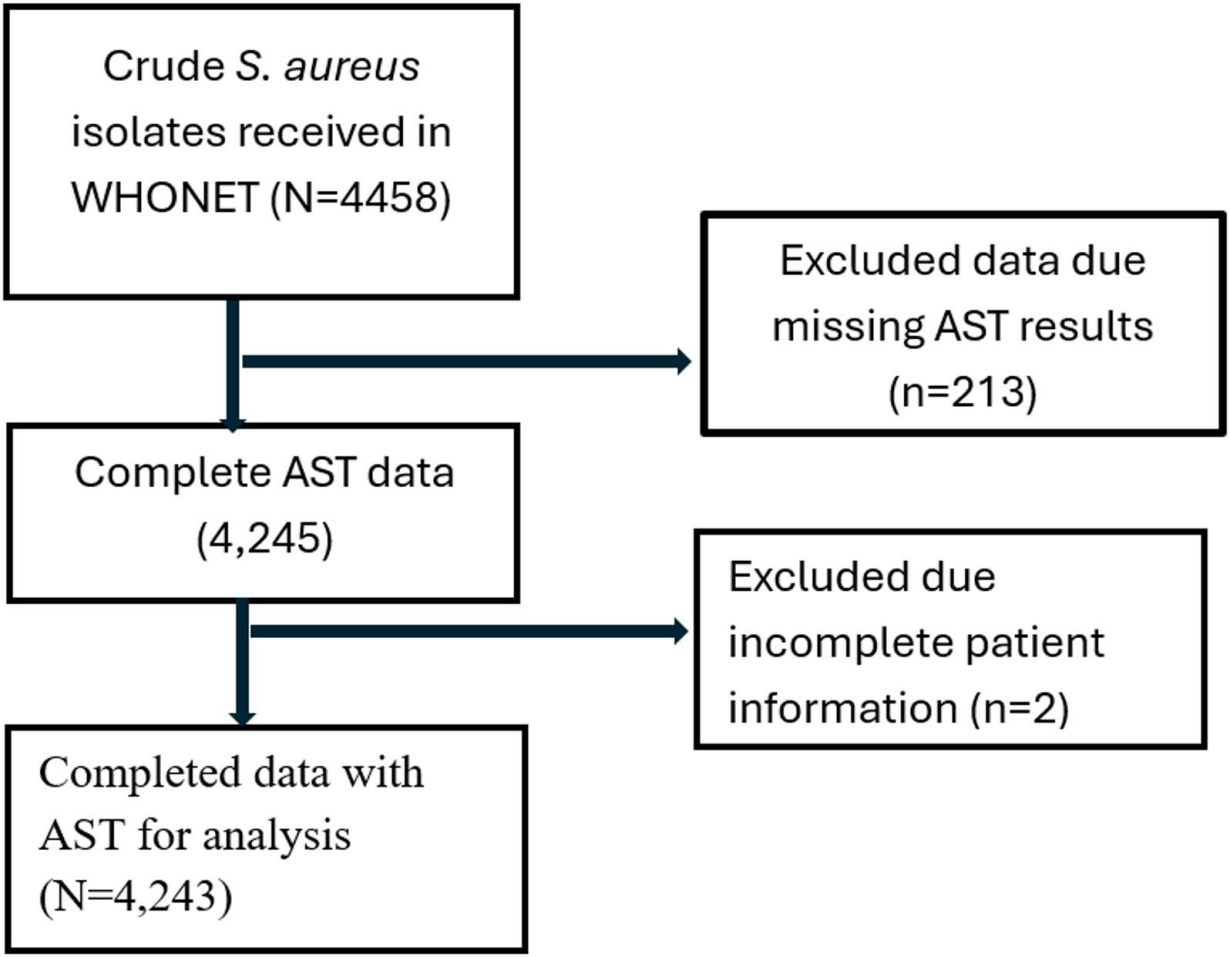
Data extraction methods.

Socio-demographic information, clinical history, hospitalization, and antibiotic use were all gathered using a standard questionnaire. An Excel sheet was used to compile all species and antimicrobial susceptibility test data, which was then exported into SPSS version 22. Crude data was extracted from WHONET and cleaned for completeness. Missed AST results for 213 patients and incomplete patient demographics for 2 datasets were excluded in the analysis (Fig. 2).

### Staphylococcus identification methods

In this study, *S. aureus* isolates were identified from clinical specimens using a series of phenotypic techniques such as Gram-stained smears, catalase and coagulase tests, and colony morphology on different media (such as mannitol salt agar and sheep blood agar). Automated systems such as BD Phoenix and Vitek® 2 (bioMérieux) were employed if the specimen was compatible and the required consumable materials were available.

### Antimicrobial susceptibility testing

The antimicrobial susceptibility testing of *S. aureus* isolates was performed with the conventional Kirby-Bauer disc diffusion method, and depending on the availability, other automated methods such as BD Phoenix and Vitek 2 were employed to generate the minimum inhibitory concentration (MIC). The results were interpreted according to the Clinical Laboratory Standard Institute (CLSI) M100 breakpoints updated each year^13^. As per the guideline recommendations and local availability, there were 14 antimicrobial panel tests that were broadly classified into a four-tier system. Antimicrobial agents classified under Tier 1 include azithromycin (15 μg) or erythromycin (15 μg), clindamycin (2 μg), cefoxitin (30 μg), tetracycline (30 μg), trimethoprim-sulfamethoxazole (1.25/23.75 μg), vancomycin (MIC), and nitrofurantoin (300 μg); Tier 2: penicillin G (10 μg) and linezolid (30 μg); and Tier 4: ciprofloxacin (5 μg) or levofloxacin (10 μg), gentamicin (10 μg), and chloramphenicol (30 μg). There was no test report of antimicrobial agents under Tier 3 across all laboratories due to the unavailability of the disc. Cefoxitin was tested as a surrogate for methicillin (oxacillin) susceptibility. A disk diffusion zone of cefoxitin measuring less than 21 mm after 24 h of incubation was interpreted as methicillin (oxacillin) resistance that confirms the presence of MRSA (14).

To ensure uniform and consistent AST reporting throughout the AMR surveillance system, experts from the national reference laboratory, in conjunction with the stewardship committee, developed a concise job aid for antibiotic selection that incorporates breakpoints according to specimen type from the M100 CLSI guideline, which was distributed to all sites. The efficacy of this adherence was assessed via ongoing mentorship. The surveillance system incorporates a confirmatory testing protocol at the national clinical bacteriology and mycology reference laboratory for randomly selected isolates of targeted pathogens, including *S. aureus,* using the available AST methods, either disc diffusion or an automated system.

### Statistical analysis

Patient-level data were captured using WHONET 2025 software version 25.15.5 (URL www.WHONET.org), which was configured with key variables such as patient demographics, patient location, clinical presentation, specimen type, current diagnosis, type of admission unit, admission status, organism identification, and antimicrobial susceptibility testing results. All isolates and patient metadata were compiled in Microsoft Excel and exported to SPSS version 22 for statistical analysis. Descriptive statistics, including cross tabulations, frequency distributions, and proportions, were used to assess the trend of antimicrobial resistance.

### Quality assurance

The national bacteriology and mycology reference laboratory has been accredited for ISO 15,189 and has a regular internal and external quality control system. Before inoculating with a specimen, all media and tube biochemicals were prepared in compliance with the manufacturer’s instructions, and their quality was assessed and recorded. The quality of antibiotic discs was evaluated and verified initially and then switched to a routine weekly internal quality control system using *Escherichia coli* ATCC 25,922, *Staphylococcus aureus* ATCC 25,923, and *Pseudomonas aeruginosa* ATCC 27,853. The discs were kept at a suitable temperature, usually between 2 and 8°C, and desiccants were used to maintain the humidity.

## Results

### Patient demographics and regional distribution of *S. aureus* isolates

Among 4,458 non-duplicated *S. aureus* isolates from various clinical specimens identified in 16 AMR sentinel sites, 4,243 of the isolates have had complete information with full antimicrobial susceptibility testing during 2020–2024. The analysis of the large population dataset revealed that males comprised 57.5% (2,439/4,243) and females 42.5% (1,804/4,243). The largest age group was infants under 1 year, 20.6% (872/4243), followed by those aged 15–24 years, 15.5% (659/4243); 5–14 years and 25–34 years comprise 14.2% (601/4243), while the elderly population (≥ 65 years) accounted for only 3.6% (153/4243), as shown in Table 1.

**Table 1:** Patient demographics and regional distribution of *S. aureus* isolated from 2020–2024.

Variable	Category	Frequency of isolates (%)
Study population	Hospital patients	4243(100)
Sex	Male	2439(57.5)
	Female	1804(42.5)
Age group (years)	< 1	872(20.6)
	1–4	545(12.8)
	5–14	601(14.2)
	15–24	659(15.5)
	25–34	601(14.2)
	35–44	380(9.0)
	45–54	247(5.8)
	55–64	185(4.4)
	65–74	101(2.4)
	75–84	43(1.0)
	85 +	9(0.2)
Regional distribution	Addis Ababa (EPHI and 5 Hospitals) Hospitals)	1978(46.6)
	Oromia (4 Hospitals)	834(19.6)
	Amhara (3 Hospitals)	702(16.5)
	Sidama (1 Hospital)	281(6.6)
	Tigray (1 Hospital)	132(3.1
	Harari (1Hospital)	306(7.2)
Infection source	Inpatient	2751(64.8)
	Outpatient	1492(35.2)
Specimen type	Pus, wound, and tissue	2287(53.9)
	Body fluid and aspirate	225(5.3)
	CSF	80(1.9)
	Blood	1083(25.5)
	Ear and eye	271(6.4)
	Respiratory	71(1.7)
	Urine	226(5.3)
Methicillin susceptibility	MRSA	712(32.2)
	MSSA	1499(67.8)

Between 2020 and 2024, a total of 4,243 *S. aureus* isolates were culture-isolated from 16 AMR sentinel sites throughout Ethiopia. Regional contributions show a concentration of reporting in urban and referral regions. Addis Ababa, which includes the National Reference Laboratory (EPHI) and five hospitals, had the highest share with 1,978 isolates (46.6%). Oromia regional state, with four hospitals, had 834 isolates (19.6%); Amhara regional state, with three hospitals, had 702 isolates (16.5%); Harari regional state, with one hospital, had 306 isolates (7.2%); Sidama regional state, with one hospital, had 281 isolates (6.6%); and Tigray had 132 isolates (3.1%).

According to the patient locations, inpatient wards accounted for 2,751 isolates (64.8%) of all the isolates recovered, whereas outpatient wards accounted for 1,492 isolates (35.2%). The significant burden of *S. aureus* infections was observed in hospitalized patients, especially those in inpatient and critical care settings (Table 1).

### Age-sex distribution of patients

**Fig. 3:**
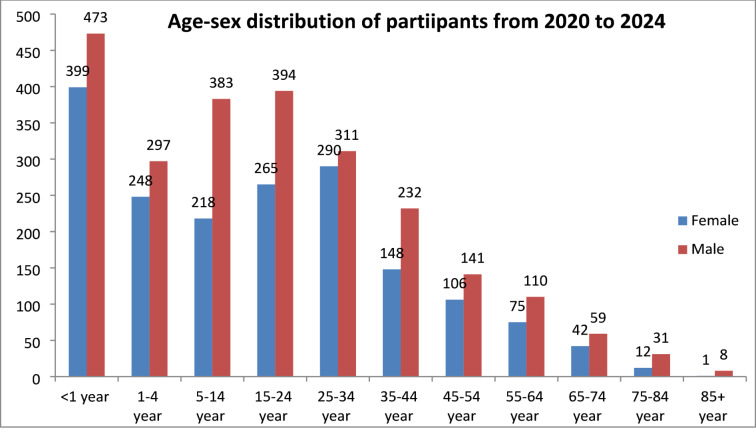
Distribution of age and sex among patients infected with *S. aureus* isolates.

Isolates of *S. aureus* from all sources of infection were high in children under five, with a male-to-female ratio of 1.2:1 (770/647). Furthermore, consistent male dominance of isolate recovery was recorded in each age group. Similarly, in 15–24 years (394 males, 365 females), 5–14 years (383 males, 218 females), and 25–34 years (311 males, 290 females), isolates were obtained. A gradual decline in the number of isolates’ distribution was observed in patients older than 35 years. A smaller number of isolates were obtained in the 75–84 years and above 85 years age groups (Fig. 3).

### *Staphylococcus aureus* among AMR sentinel hospitals

From 2020 to 2024, among 16 AMR sentinel sites, ALERT Comprehensive Specialized Hospital in Addis Ababa contributed the highest contributions, 15% (629/4243), followed by Jimma University Medical Center in Oromia, 11% (455/4243). Other major contributors included University of Gondar Hospital, 10% (435/4243); Yekatit-12 Hospital, 8% (350/4243); and Tikur Anbessa Specialized Hospital (338/4243). The least contributors were Mattu Karl Comprehensive Specialized Hospital and the APHI-Dessie branch laboratory.

Addis Ababa accounted for six sentinel sites, contributing a combined 1,978 isolates (47%), with notable increases observed at Tikur Anbessa Specialized Hospital and ALERT Hospital over the five-year period. Annual trends indicated a steady increase in the number of isolates recovered from various specimens, rising from 395 in 2020 to 1,393 in 2024. This increase was due to the expansion of surveillance capacity and improved reporting in the surveillance sites (Table 2).

**Table 2:** The distribution of *S. aureus* in 16 AMR sentinel sites of Ethiopia from 2020 to 2024.

AMR site	Number of specimens by year					Total	
	2020	2021	2022	2023	2024	n	(%)
Tikur Anbessa Specialized Hospital	9	12	70	124	123	338	8%
St. Paul’s Hospital Millennium	41	41	64	74	73	293	7%
Zewditu Memorial Hospital	0	63	78	48	52	241	6%
Yekatit-12 Hospital	0	64	87	117	82	350	8%
National Reference Laboratory	19	20	35	31	22	127	3%
ALERT Comprehensive Hospital	0	50	133	186	260	629	15%
Jimma University Medical Center	102	54	108	96	95	455	11%
Nekemte Referral Laboratory	0	0	20	111	115	246	6%
Adama Referral Laboratory	0	8	15	26	70	119	3%
Mattu Karl Comprehensive Specialized Hospital	0	0	0	0	24	24	1%
University of Gondar Hospital	112	64	119	51	89	435	10%
Felege Hiwot Hospital	0	27	61	56	23	167	4%
APHI-Dessie Branch Laboratory	1	0	11	34	54	100	2%
Hawassa University Hospital	93	34	17	74	63	281	7%
Ayder Hospital (AyCSH)	18	0	0	33	81	132	3%
Hiwot Fana Hospital	0	0	10	129	167	306	7%
Total	395	437	828	1190	1393	4243	100%

### Distribution of *S. aureus* in clinical samples

**Fig. 4:**
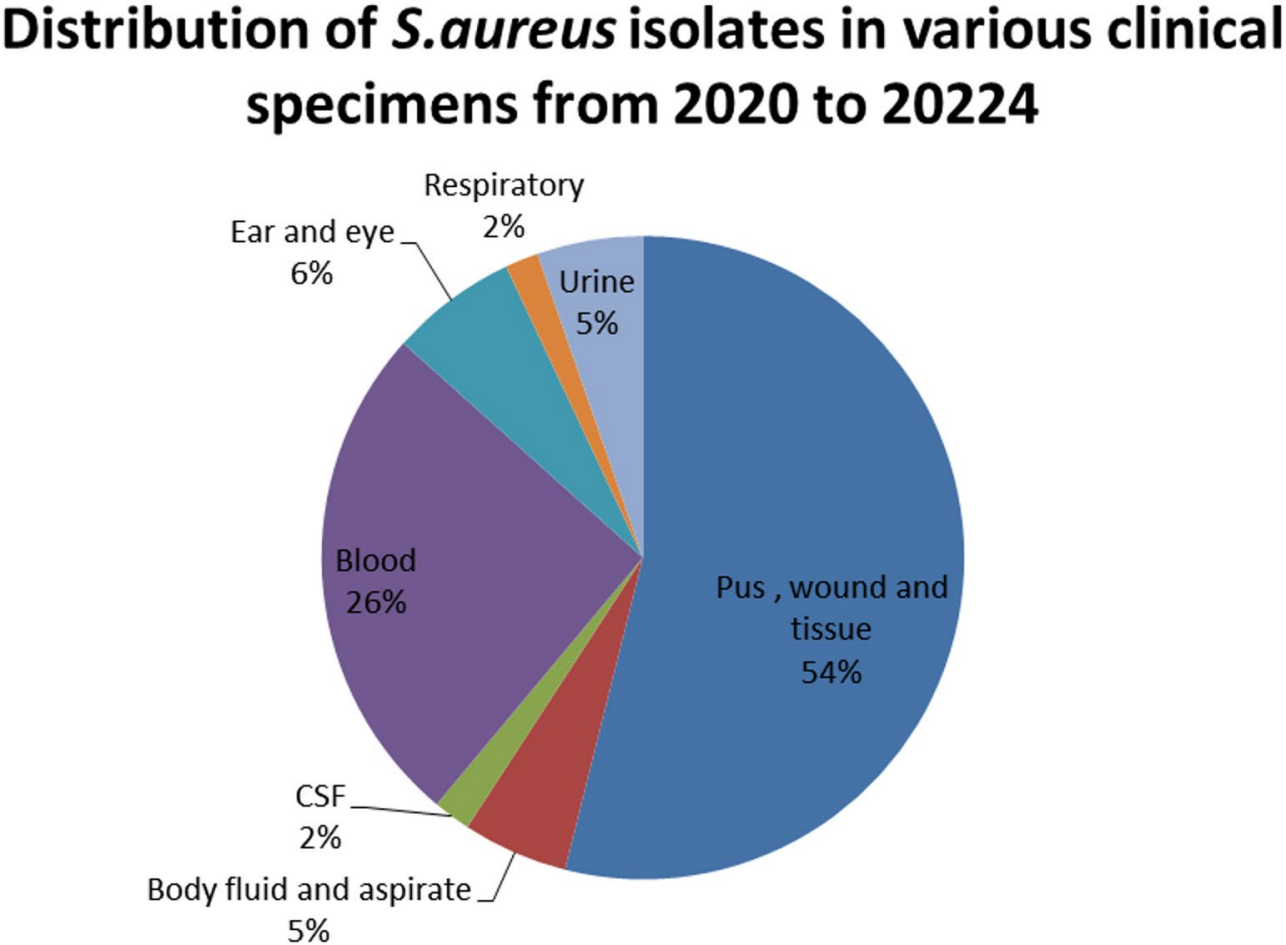
Distribution of *S. aureus* in clinical samples collected from 2020 to 2024.

A total of 4,243 *S. aureus* isolates were recovered from various clinical specimens between 2020 and 2024. The majority were isolated from pus samples, accounting for 2,287 isolates (54%)**,** followed by blood specimens with 1,083 isolates (26%). Other notable sources included ear and eye swabs (271, 6%), urine samples (226, 5%), and body fluids (225, 5%), contributing to the total isolates. Less frequently sampled specimens included cerebrospinal fluid (80, 2%), and respiratory samples (71, 2%) were also among the contributors. The predominance of pus and blood specimens reflects the clinical burden of *S. aureus*-associated skin, soft tissue, and bloodstream infections in the surveillance population. The diversity of specimen types also highlights the organism’s capacity to cause infections across multiple anatomical sites (Fig. 4).

### Antimicrobial susceptibility pattern of the isolates

Over the five-year laboratory-based AMR surveillance period, *S. aureus* isolates were tested against antibiotics that showed variable susceptibility patterns. Most of the isolates were consistently highly resistant to penicillin in the range of 89% to 91%; only about 10% of the isolates were susceptible. The susceptibility of methicillin (oxacillin) was 78% in 2020 and decreased to 63% in 2021–2022, then 68–70% from 2023 to 2024, which showed a variable pattern of methicillin susceptibility. On the other hand, gentamicin was susceptible to 89% in 2020, 70–77% in 2021 to 2022, and 81–84% in 2023 to 2024. Similarly, ciprofloxacin increased susceptibility from 85 to 87% from 2021 to 2022; however, it showed decreased susceptibility from 81 to 70% from 2022 to 2024. Last-line antibiotics like vancomycin (95% in 2020, increased to 98% in 2023, and 100% in 2024) and linezolid (95% in 2024) have maintained their effectiveness and were included in the last year of the analysis (Table 3).

**Table 3:** Antimicrobial susceptibility pattern of *S*. *aureus* isolates from 2020 to 2024.

Antimicrobial agent	2020			2021			2022			2023			2024		
	*R%*	*I%*	*S%*	*R%*	*I%*	*S%*	*R%*	*I%*	*S%*	*R%*	*I%*	*S%*	*R%*	*I%*	*S%*
Penicillin G	90%	0%	10%	91%	0%	9%	90%	0%	10%	89%	0%	11%	89%	0%	11%
Oxacillin	22%	0%	78%	37%	0%	63%	37%	0%	63%	32%	0%	68%	30%	0%	70%
Gentamicin	11%	0%	89%	26%	3%	70%	22%	2%	77%	17%	1%	81%	16%	0%	84%
Ciprofloxacin	10%	4%	85%	12%	1%	87%	16%	3%	81%	25%	5%	70%	22%	7%	71%
Levofloxacin	–	–	–	–	–	–	–	–	–	–	–	–	25%	5%	70%
SXT	31%	4%	64%	31%	3%	66%	25%	5%	71%	25%	4%	71%	25%	4%	71%
Clindamycin	13%	3%	84%	16%	3%	81%	14%	4%	83%	20%	5%	75%	18%	5%	77%
Azithromycin	38%	2%	60%	33%	5%	62%	28%	3%	70%	45%	3%	52%	38%	3%	59%
Erythromycin	34%	8%	58%	34%	11%	55%	32%	7%	60%	36%	10%	53%	38%	11%	52%
Nitrofurantoin	–	–	–	–	–	–	–	–	–	20%	3%	77%	22%	8%	69%
Linezolid	–	–	–	–	–	–	–	–	–	–	–	–	5%	3%	92%
Vancomycin	5%	0%	95%	–	–	–	–	–	–	2%	0%	98%	0%	0%	100%
Chloramphenicol	4%	1%	94%	–	–	–	7%	2%	91%	10%	2%	88%	7%	2%	92%
Tetracycline	38%	3%	59%	49%	6%	46%	32%	8%	61%	39%	4%	57%	40%	5%	55%

SXT; Trimethoprim/Sulfamethoxazole.

Antimicrobial agents showed ≥ 80% susceptibility rates, including gentamycin (2020, 2023, and 2024); ciprofloxacin (2020, 2021, and 2023); clindamycin (2020–2023); vancomycin; and chloramphenicol, which were effective for empirical treatment during the tested years (Table 3).

### Trend of antimicrobial resistance

**Fig. 5:**
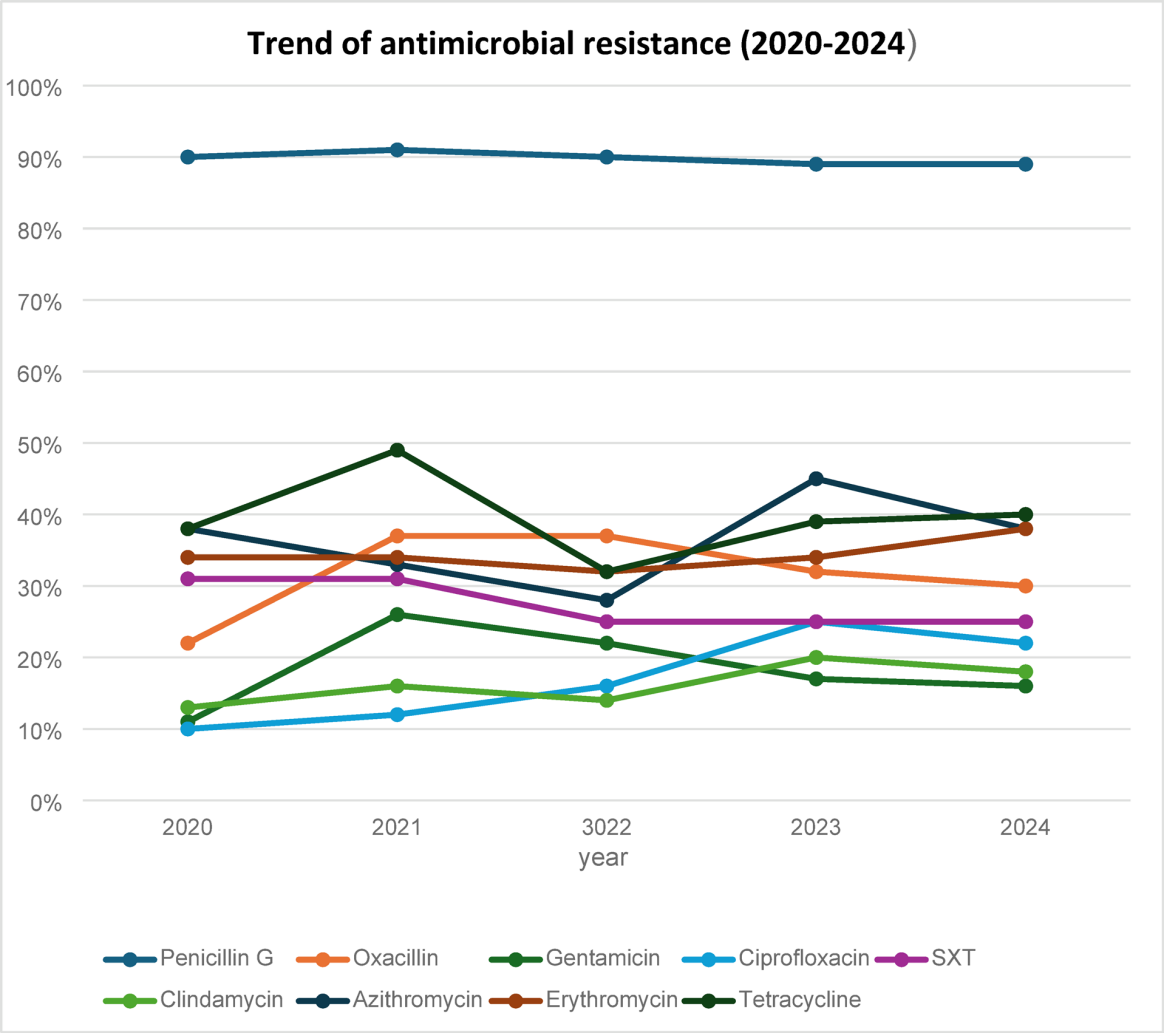
National trend of antimicrobial resistance among *S. aureus* isolates collected from 2020 to 2024.

The trend of penicillin resistance among tested isolates showed persistently high (~ 90%) between 2020 and 2024. The proportion of methicillin-resistant *S. aureus* (MRSA) showed noticeable variation over the five-year period. Among tested isolates, the overall prevalence of MRSA was 32.2% (95% CI 30.3, 34.2). About 22% of the isolates in 2020 were MRSA, which increased dramatically to 37% in 2021 and 2022. MRSA rates, nevertheless, decreased after that, falling to roughly 32% in 2023 and 30% in 2024. Overall, the pattern suggests an initial increase in MRSA prevalence followed by a slight decline. Gentamicin resistance has decreased since 2021 from 26 to 16% in 2024. Similarly, SXT resistance has decreased from 31% in 2020 to 25% in 2024 (Fig. 5).

### Trend of methicillin-resistant *Staphylococcus aureus* by hospital wards

**Fig. 6:**
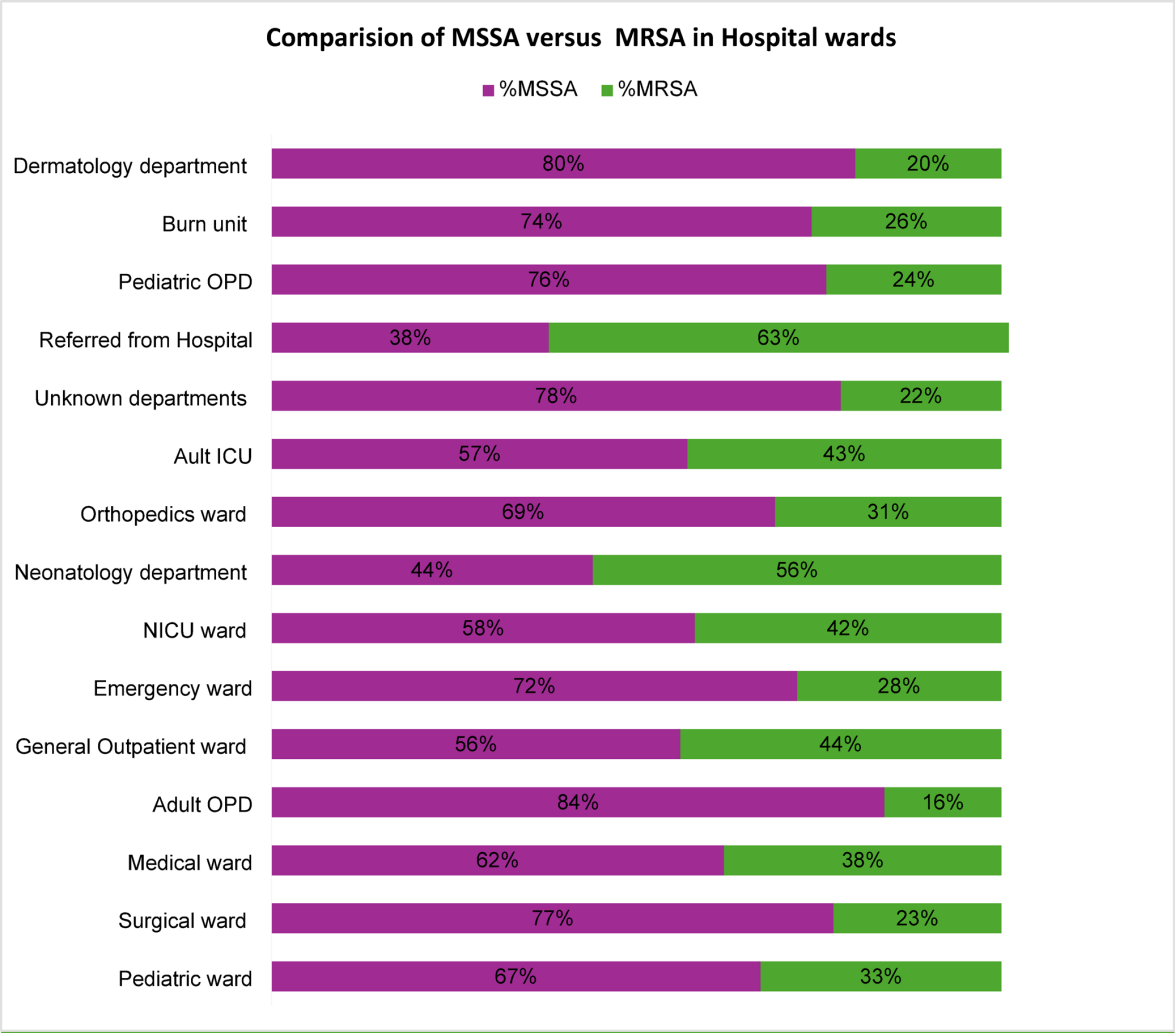
Methicillin susceptibility pattern of *S. aureus* in various words.

Methicillin susceptibility patterns among hospital wards showed varied resistance rates. Compared to MSSA isolates, MRSA was observed in patients transferred from other hospitals (63%), neonatal departments (56%), outpatient departments (44%), and NICU (42%). The lowest MRSA was observed in isolates of Adult OPD (16%), Dermatology (20%), and the Burn Unit (26%). The comparison of MRSA and MSSA across hospital wards (Fig. 6).

## Discussion

*Staphylococcus aureus* is the most frequently isolated pathogen causing community- and healthcare-associated infections. This retrospective laboratory-based AMR surveillance study, which analyzes isolates obtained from sixteen sentinel sites across Ethiopia, offers thorough insights into the pattern and trend of antimicrobial resistance of *S. aureus* over a period of five years. The rising prevalence of *S. aureus* infection within hospitals that are part of the national AMR surveillance system is reflected in the increased specimen flow and high isolation rate in various clinical specimens.

The present analysis demonstrated that most *S. aureus* isolates were obtained from male patients (57.5%), and a high number of isolates were identified in the under-five-years-of-age group (33.4% (1417/4243)), while a fewer number of isolates were from patients 65 years and above (3.6% (153/4243)). This demographic distribution is consistent with findings from previous Ethiopian studies; in Gondar, male dominance was reported at 55.5%^15^, in Debre-Markos, 57.9%^16^, and in Rio de Janeiro, Brazil, 58%^17^. Another local study from various hospitals of the Amhara regional state has also documented a comparable isolation rate (36.7%) in children under five^18^. Recent studies supported that the possible reason for male dominance over females in bacterial infections includes the impact of testosterone in enhancing *S. aureus* virulence^19^ and the protective effect of the female estrogen hormone in bacterial colonization from the GI tract and the female reproductive system^20,21^.

In the present study, many of the isolates were from pus, wound, and tissue samples (54%) and blood (25.5%) specimens. This is consistent with earlier research at Yekatit 12 Hospital in Addis Ababa that documented a 55.1% isolation rate from pus^22^. It is also comparable to a seven-year study in Vietnam where half of the isolates were from pus specimens^23^.

The trend of penicillin-resistant *S. aureus* over the five-year period was constantly high at 89–91%, which is in line with previous similar studies: Addis Ababa, 86.7%^24^; and Hawassa, Southern Ethiopia, 91%^25^. It also extends concordantly with the global pattern, which has been universal for decades due to more than 90% of staphylococcal isolates producing β-lactamase^2,26^, which hydrolyzes the β-lactam ring in β-lactam antibiotics to render them inactive.

In our study, the overall prevalence of MRSA was 32.2%, which is in line with the pooled prevalence of 32.5% reported in a meta-analysis conducted in Ethiopia^27^ and comparable to the China Antimicrobial Resistance Surveillance System’s 29.1%^28^ and the Global Antimicrobial Surveillance System (GLASS) report’s 27.1%^29^; however, it is lower as compared to the previous studies in Ethiopia, 57.1%^30^, and in neighboring countries Kenya, 53.4%^31^; Eritrea, 72%^32^; Vietnam, 73.1%^33^; and Nepal, 70.6%^34^. This noticeable difference could be attributed to the difference in study population, number of isolates, and infection prevention practice. On the other hand, the result from this study was higher than previous reports from Addis Ababa, Ethiopia, 7.5%^35^, and Hawassa, 17.9%^36^; this might be due to the difference of sample sources, in which our study includes only patients in contrast to the inclusion of colonization samples.

Nationally, the trend of MRSA slightly declined from 37% in 2021 to 30% in 2024, but its burden is markedly high in various hospital wards. The highest resistance rate was recorded in patients transferred from other hospitals (63%), followed by neonatal departments (56%), outpatient departments (44%), and NICU (42%), which was comparable with the study in Saudi Arabia, where high MRSA was found in inpatient settings, particularly in surgical (56.0%), ICU (53.4%), and medical wards (52.7%)^37^. A high rate of outpatient MRSA strongly indicates that there is growing community-acquired MRSA. On the other hand, the trend was the opposite pattern compared to the study in Lebanon, which showed a steady rise from 34.4% in 2017 to 43.2% in 2023 in a seven-year period^38^. This might be attributable to the effect of AMR surveillance awareness in prudent use of antimicrobials.

The trend of gentamicin resistance has decreased since 2021 from 26 to 16% in 2024. Similarly, SXT has decreased from 31% in 2020 to 25% in 2024, which was a similar pattern to a six-year trend analysis in China that showed that the resistance rates of SXT and gentamicin were declining annually^39^. This might be due to sparingly and sensibly staying at a steady, low level of aminoglycoside and trimethoprim-sulfamethoxazole consumption in the treatment of the infection.

Lastly, the resistance level of last-resort antibiotics against MRSA, including vancomycin and linezolid, remains below 5%, and a similar trend has been reported in India^40^, but the trend of vancomycin resistance is slightly decreased compared with the previous five-year analysis in Ethiopia, 5.1%^24^. This difference might be due to increased adherence to the antimicrobial susceptibility testing method of vancomycin with MIC rather than disc diffusion, as recommended by CLSI. Moreover, mentorships and training on antimicrobial susceptibility testing, antimicrobial stewardship, diagnostic stewardship, and IPC among healthcare professionals improved prudent use of antimicrobials in line with the national treatment guidelines.

### Limitation of the study

Even though continuous mentorship and supervision have substantially improved the test capacity of the AMR surveillance system in Ethiopia during a five-year period, there were some limitations to be considered in this study. Since the study was retrospective, data cleaning at study sites was not thoroughly undertaken before being sent to EPHI. As a result, some patient information received from sentinel sites through WHONET was insufficient for analysis, with inconsistent and missing AST data due to stockouts at some sentinel sites. The study made no distinction between MRSA obtained in hospitals and MRSA acquired in the community, both of which have significant public health implications. Due to a paucity of supplies, additional beta-lactamase and molecular testing to detect resistance genes were not performed.

## Conclusions

The analysis demonstrates that *Staphylococcus aureus* remains a major cause of clinical infections in Ethiopia, affecting all age groups but disproportionately affecting infants, inpatients, and patients in high-risk wards such as neonatal and adult ICUs.

The predominance of its isolation from pus and blood specimens highlights the organism’s significant role in soft tissue and bloodstream infections. Although there has been a slight decline trend of MRSA since 2022, the persistently high trend of penicillin-resistant *S. aureus* and fluctuating resistance rates in routinely tested antimicrobials underscore ongoing challenges in clinical management. The susceptibility of *S. aureus* isolates to last resort antibiotics, vancomycin and linezolid, remains promising for treatment. The significant presence of MRSA in the participating hospitals warrants the need for continuous surveillance and implementation of appropriate infection prevention and control measures at these facilities.

## Data Availability

All data generated or analyzed during this study are included in this published article.

## Abbreviations

AMR: Antimicrobial resistance
AST: Antimicrobial susceptibility testing
CLSI: Clinical Laboratory Standards Institute
GLASS: Global Antimicrobial Surveillance System
ICU: Intensive care unit
IPC: Infection prevention and control
MRSA: Methicillin-resistant *Staphylococcus aureus*
MSSA: Methicillin-susceptible *Staphylococcus aureus*
VRSA: Vancomycin-resistant *Staphylococcus aureus*
WHONET: World Health Organization Networking
